# Corrupted ER‐mitochondrial calcium homeostasis promotes the collapse of proteostasis

**DOI:** 10.1111/acel.13065

**Published:** 2019-11-12

**Authors:** Zahra Ashkavand, Shaarika Sarasija, Kerry C. Ryan, Jocelyn T. Laboy, Kenneth R. Norman

**Affiliations:** ^1^ Department of Regenerative and Cancer Cell Biology Albany Medical College Albany NY USA

**Keywords:** Alzheimer's disease, *Caenorhabditis elegans*, calcium homeostasis, mitochondria, oxidative stress

## Abstract

Aging and age‐related diseases are associated with a decline of protein homeostasis (proteostasis), but the mechanisms underlying this decline are not clear. In particular, decreased proteostasis is a widespread molecular feature of neurodegenerative diseases, such as Alzheimer's disease (AD). Familial AD is largely caused by mutations in the presenilin encoding genes; however, their role in AD is not understood. In this study, we investigate the role of presenilins in proteostasis using the model system *Caenorhabditis elegans*. Previously, we found that mutations in *C. elegans* presenilin cause elevated ER to mitochondria calcium signaling, which leads to an increase in mitochondrial generated oxidative stress. This, in turn, promotes neurodegeneration. To understand the cellular mechanisms driving neurodegeneration, using several molecular readouts of protein stability in *C. elegans*, we find that presenilin mutants have widespread defects in proteostasis. Markedly, we demonstrate that these defects are independent of the protease activity of presenilin and that reduction in ER to mitochondrial calcium signaling can significantly prevent the proteostasis defects observed in presenilin mutants. Furthermore, we show that supplementing presenilin mutants with antioxidants suppresses the proteostasis defects. Our findings indicate that defective ER to mitochondria calcium signaling promotes proteostatic collapse in presenilin mutants by increasing oxidative stress.

## Graphical abstract

Ashkavand et al uncover a critical role of ER‐mitochondrial calcium homeostasis in the regulation of cellular proteostasis. Mutations that increase ER to mitochondrial calcium signaling lead to increased oxidative stress which promotes proteostatic collapse.

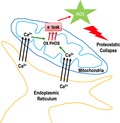

## 1 INTRODUCTION

The maintenance of a highly functional proteome is critical for organismal health. This is particularly important in the aging nervous system. Indeed, reduced protein homeostasis (proteostasis) is a widespread molecular aspect of neurodegeneration, but the mechanisms underlying this decline are not clear. Alzheimer's disease (AD) is the most prevalent neurodegenerative disease and is associated with the accumulation of protein aggregates, such as amyloid beta and neurofibrillary tangles in the brain of patients (Citron, 2002). Familial AD (FAD) is largely caused by mutations in the presenilin encoding genes. However, despite the identification of presenilin's association with AD over 20 years ago, their role in AD is not understood (Kelleher & Shen, 2017;Selkoe & Hardy, 2016). Presenilins are widely expressed predominantly endoplasmic reticulum (ER) membrane proteins that form the proteolytic subunit of the gamma secretase, which is an integral membrane protein complex that cleaves single‐pass transmembrane proteins (De Strooper, Iwatsubo, & Wolfe, 2012;Smolarkiewicz, Skrzypczak, & Wojtaszek, 2013). Previously using *Caenorhabditis elegans* as a model system to understand presenilin function, we found that mutations in the *C. elegans* presenilin gene (*sel‐12*) cause elevated ER to mitochondria calcium signaling, which leads to an increase in mitochondrial calcium content that results in increased mitochondrial oxidative phosphorylation and electron leak accelerating oxidative stress (Sarasija et al., 2018). This, in turn, promotes neurodegeneration (Sarasija et al., 2018). To help understand the cellular mechanisms driving neurodegeneration, here, we have utilized multiple transgenic models expressing metastable proteins to evaluate protein homeostasis in *sel‐12* mutants. Remarkably, we found widespread defects in proteostasis in *sel‐12* mutants expressing these metastable proteins. Furthermore, we utilized heat stress to destabilize endogenous protein folding and examined the ability of *sel‐12* animals to recover from this insult. Strikingly, *sel‐12* mutants are highly susceptible to heat stress and, unlike wild‐type animals, cannot recover from acute high temperature exposure. Additionally, we found if we reduced ER to mitochondrial calcium signaling in *sel‐12* mutants, we could significantly prevent the proteostasis defect observed in *sel‐12* mutants expressing metastable proteins and improve the survival of *sel‐12* mutants after acute heat stress. Moreover, we found that supplementing *sel‐12* mutants with antioxidants could also suppress these proteostasis defects. Our findings indicate that defective ER to mitochondria calcium signaling promotes proteostatic collapse in *sel‐12* mutants by increasing oxidative stress.

## 2 RESULTS

### 2.1 SEL‐12/presenilin is required for proteostasis

To investigate the status of protein homeostasis in *sel‐12* mutants, we utilized several transgenic animals that express ectopic metastable and aggregation‐prone proteins. These include (a) body wall muscle expression of polyglutamine (polyQ) construct Q35::YFP (*rmIs132*) which has been shown to progressively aggregate as the transgenic animals age (Morley, Brignull, Weyers, & Morimoto, 2002), (b) body wall muscle expression of human amyloid beta1‐42 (Abeta1‐42)(*dvIs100*) which has been demonstrated to aggregate and promote progressive locomotory defects in transgenic animals as they age (McColl et al., 2012), and (c) pan‐neuronal expression of a human pathogenic V337M mutant tau protein (*bkIs10*) which, similar to the Abeta1‐42 strain, has been shown to aggregate and promote progressive locomotory defects as the transgenic animals age (Kraemer et al., 2003).

**Figure 1:**
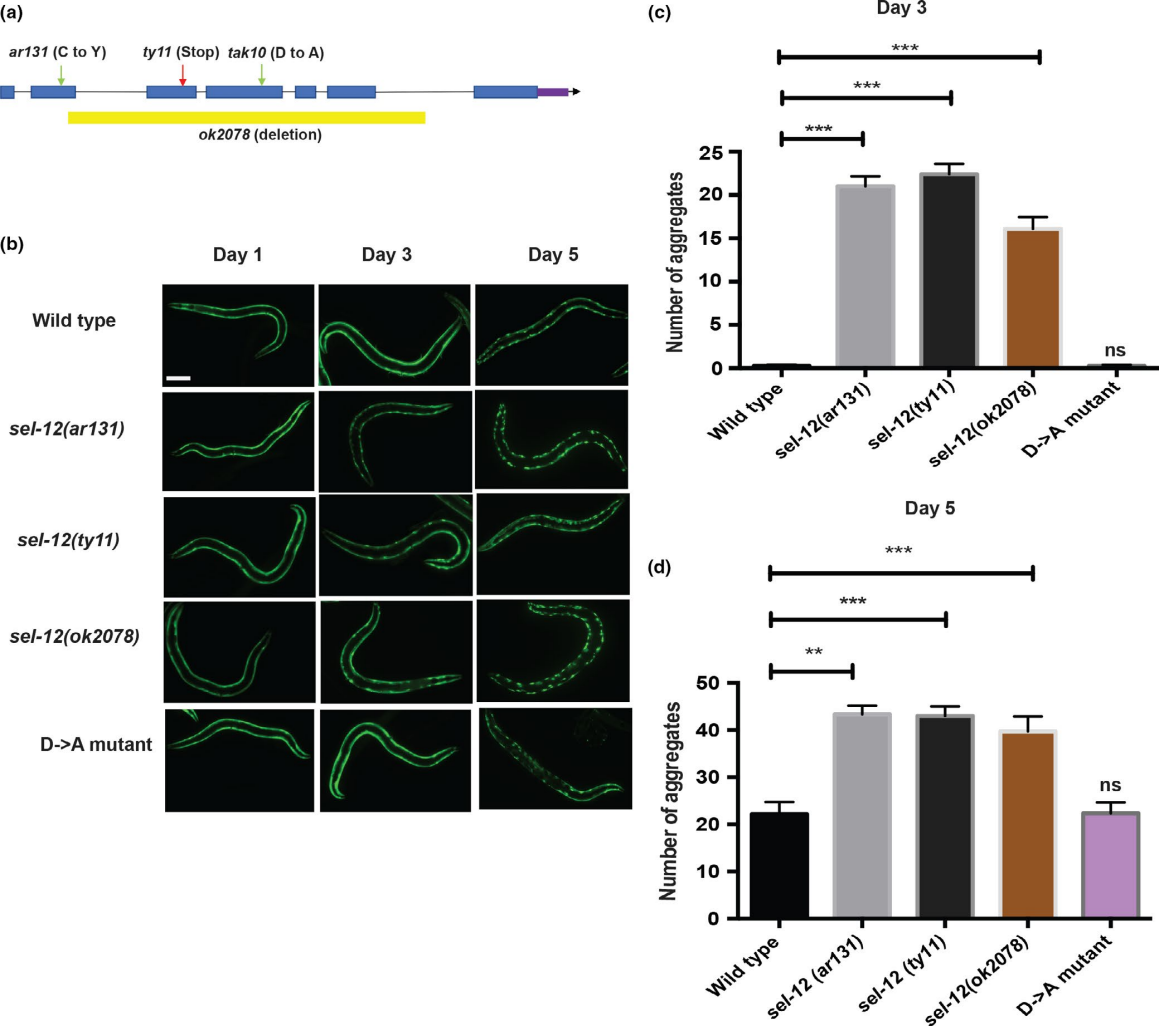
Premature aggregation of Q35::YFP in *sel‐12* mutants is independent of gamma‐secretase protease activity. (a) Schematic representation of *sel‐12* locus indicating the location of the mutation in the *sel‐12* mutants analyzed in this study. (b) Representative images of wild‐type, *sel‐12 (ar131), sel‐12 (ty11)*, *sel‐12(ok2078)*, and D‐>A mutants expressing Q35::YFP (*rmIs132*) at day 1, day 3, and day 5 adulthood. Scale bar indicates 100 μm. (c and d) Quantification of Q35::YFP aggregation in wild‐type and *sel‐12(ar131), sel‐12(ty11)*, *sel‐12(ok2078)*, and *sel‐12(D‐>A)* animals at day 3 and day 5 adulthood (*N* = 30 animals per group). Data are displayed as mean ± *SEM*, and all comparisons have been made to wild‐type animals or indicated strains. ns *p* > .05, **p < .01, and ***p < .001 were determined using one‐way ANOVA

As previously shown (Morley et al., 2002), we have found that the expression of Q35::YFP in day 1 and day 3 wild‐type adult animals is soluble and is evenly distributed in muscle cells (Figure 1b,c). However, when these animals reach day 5 of adulthood, aggregation of Q35::YFP is readily apparent (Figure 1b,c). We introduced this transgene into three *sel‐12* mutants, *sel‐12(ar131)*, *sel‐12(ok2078),* and *sel‐12(ty11)*. *sel‐12(ar131)* mutants carry a missense mutation in the *sel‐12* gene that changes a cysteine to a tyrosine (C60Y), which is a conserved change observed in human presenilin that is associated with FAD (Levitan & Greenwald, 1995), *sel‐12(ok2078)* mutants have a large deletion of the *sel‐12* locus and *sel‐12(ty11)* mutants contain a premature stop codon in the *sel‐12* open reading frame (Cinar, Sweet, Hosemann, Earley, & Newman, 2001) (Figure 1a). From the analysis of the *sel‐12* mutants as day 1 adults, we found that they resemble wild‐type animals showing an even distribution of Q35::YFP (Figure 1b). However, in contrast to wild‐type animals, day 3 adult *sel‐12* mutants show a striking premature accumulation of Q35::YFP aggregates that progresses further by day 5 (Figure 1b,c). This precocious aggregation of Q35::YFP suggests *sel‐12* mutants have defects in proteostasis.

**Figure 2:**
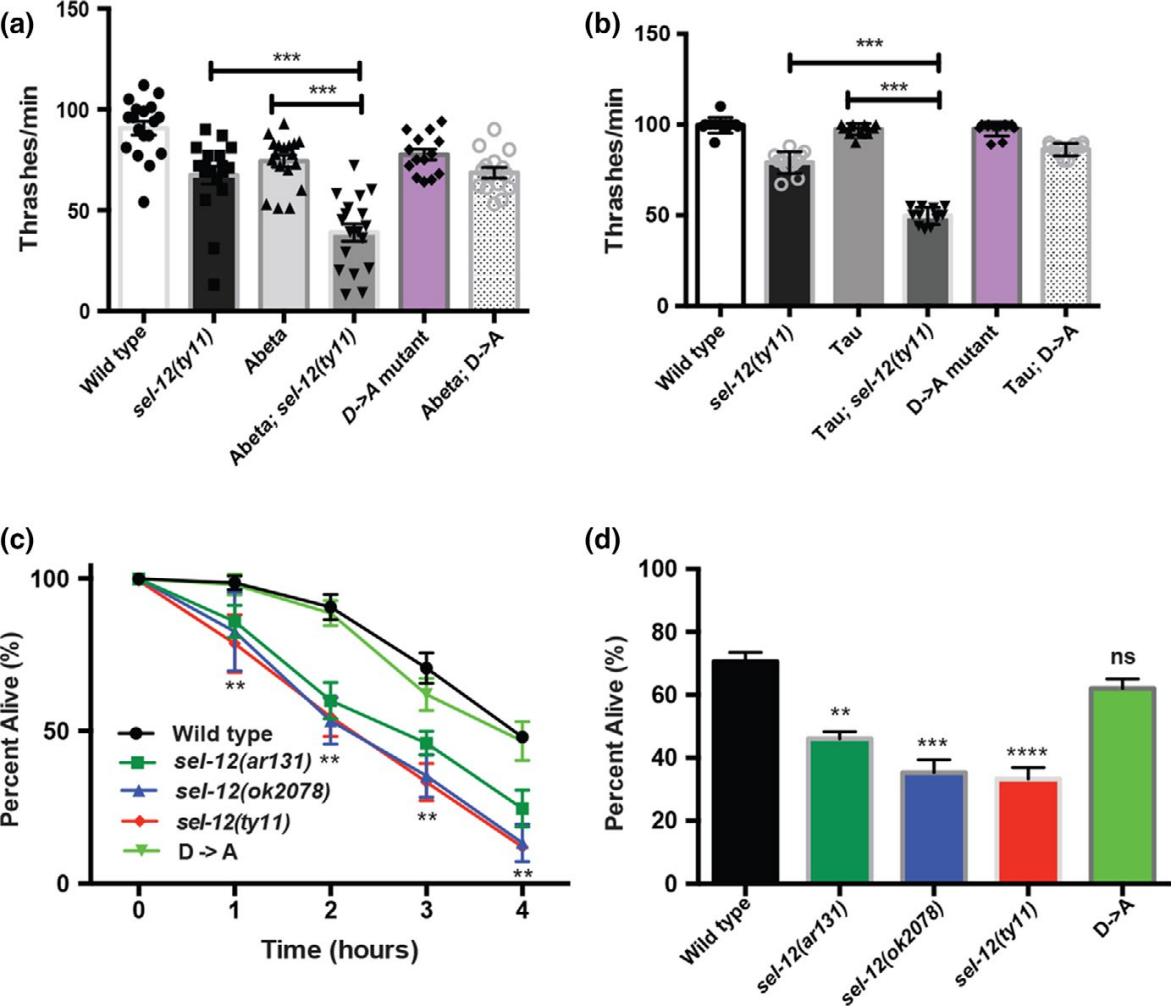
Proteostatic collapse in *sel‐12* mutants is independent of gamma‐secretase protease activity. (a) Swimming assay of wild‐type, *sel‐12(ty11),* and *sel‐12(D‐>A)* animals expressing human Abeta1‐42 (*dvIs100*) was conducted by measuring the number of body bends in liquid (*N* = 20 animals per group). (b) Swimming assay of wild‐type, *sel‐12(ty11),* and *sel‐12(D‐>A)* animals expressing human V337M mutant tau were conducted by measuring the number of body bends in liquid (*N* = 20 animals per group). (c) Survival of day 1 animals after exposure to heat stress at 37°C for 1–4 hr (50 animals per group, done in triplicate). (d). Survival of day 1 mutants after exposure to heat stress at 37°C for 3 hr (50 animals per group, done in triplicate). Data are displayed as mean ± *SEM*, and all comparisons have been made to wild‐type animals or indicated strains. ns *p* > .05, **p < .01, and ***p < .001 were determined using one‐way ANOVA (a, b, and c) and two‐way ANOVA (c)

To further investigate proteostasis in *sel‐12* mutants, we next analyzed animals expressing human Abeta1‐42. Previous studies have shown that heterologous expression of human Abeta1‐42 in the body wall muscle induces proteostatic stress and results in reduced motility (McColl et al., 2012). Indeed, we found that Abeta1‐42 expressing animals showed a reduction in swimming behavior compared with wild‐type animals (Figure 2a; Figure S1a). Consistent with *sel‐12* mutants having a proteostasis defect, when this transgene is introduced into the *sel‐12* mutant background, the motility observed in day 1 adult *sel‐12* animals expressing Abeta1‐42 is significantly worse compared with age‐matched *sel‐12* mutants or Abeta1‐42 expressing only animals (Figure 2a; Figure S1a). These data further suggest that *sel‐12* mutants have defects in proteostasis. Next, to investigate whether similar defects can arise in the nervous system, we analyzed animals pan‐neuronally expressing human pathogenic V337M mutant tau, which progressively aggregates and causes locomotory defects as the transgenic animals age. Consistent with previous studies (Fatouros et al., 2012;Kraemer et al., 2003), we found that wild‐type day 1 adult animals expressing mutant tau display normal motility (Figure 1d; Figure S1b). However, unlike wild‐type animals, day 1 adult *sel‐12* mutants expressing tau display a severe reduction in swim rate compared with control animals (Figure 1d; Figure S1b). Moreover, consistent with the severe reduction in motility, we find a significant elevation in axon abnormalities in day 1 adult *sel‐12* mutants expressing mutant tau unlike age‐matched wild‐type, *sel‐12,* or tau expression in wild‐type animals (Figure S1c‐e). Taken together, these data indicate that *sel‐12* animals show defects in proteostasis and are prone to protein aggregation.

Since our studies thus far investigating proteostasis in *sel‐12* mutants have involved the expression of ectopic proteins, we next sought to more specifically investigate the state of proteostasis on endogenous proteins. To accomplish this, we heat‐stressed the animals to induce protein folding defects (Zevian & Yanowitz, 2014) and investigated how well *sel‐12* mutants recover from this insult. We heat‐stressed day 1 adult wild‐type, *sel‐12(ar131)*, *sel‐12(ok2078)*, and *sel‐12(ty11)* animals at 37°C for 1, 2, 3, and 4 hr. After allowing the animals to recover from this extreme heat stress, they were examined for survival. Strikingly, the *sel‐12* mutants showed increased sensitivity to heat stress and, unlike wild‐type animals, showed a reduced rate of survival (Figure 2c,d). Collectively, these data indicate that loss of *sel‐12* function leads to increased susceptibility to proteostatic collapse.

### 2.2 Proteostasis defects in *sel‐12* mutants are independent of gamma‐secretase activity

One of the critical activities of presenilin is its role as the catalytic subunit in the gamma‐secretase complex. In addition to being critical for promoting Notch signaling, the proteolytic activity of the gamma secretase has been proposed to be important for the breakdown of membrane components, and in the absence of presenilin function, membrane proteins can accumulate (De Strooper et al., 2012). The gamma‐secretase activity of presenilin is dependent on the presence of two intact aspartate residues, D257 and D385 in human PSEN1. Disruption of one aspartate residue leaves presenilin catalytically nonfunctional (Wolfe et al., 1999). Previously, we discovered that neurodegeneration in *sel‐12* mutants does not occur due to loss of gamma‐secretase proteolytic activity. Indeed, we found that a *sel‐12* rescue construct that contains an aspartate (D) to alanine (A) mutation in SEL‐12 (D226A), which is critical for its aspartyl protease activity (equivalent to human PSEN1 D257), could fully rescue the neurodegenerative phenotypes but not phenotypes caused by loss of Notch signaling (Sarasija et al., 2018). Moreover, we also found that gamma‐secretase inhibitors do not show neurodegenerative phenotypes like *sel‐12* loss of function mutants (Sarasija et al., 2018). Thus, to test the role of SEL‐12's protease activity in proteostasis, using CRISPR/Cas9 technology, we introduced a knock‐in D to A mutation in a residue critical (D226A) for aspartyl protease function (Sarasija et al., 2018;Sarasija & Norman, 2015) in the endogenous *sel‐12* gene product (Figure 1a). First, like the *sel‐12* loss of function mutations, *sel‐12(ar131)*, *sel‐12(ok2078)*, and *sel‐12(ty11)*, *sel‐12(D‐>A)* mutants show a severe egg‐laying defect and protruding vulva consistent with loss of Notch signaling (Cinar et al., 2001;Levitan & Greenwald, 1995;Sarasija et al., 2018;Sarasija & Norman, 2015). Concordant with our previous studies, we found that the *sel‐12(D‐>A)* mutants did not show signs of neurodegeneration or mitochondria defects. Indeed, *sel‐12(D‐>A)* mutants, unlike *sel‐12(ty11)* mutants, which have an identical Notch loss of function phenotype as *sel‐12(D‐>A)* mutants, showed normal response to light touch (Figure S2a). Moreover, while *sel‐12(ty11)* mutants show elevated mitochondrial calcium loading and mitochondrial respiration, *sel‐12(D‐>A)* mutants display normal mitochondrial activity (Figure S2b‐f). Nevertheless, to investigate whether *sel‐12* protease activity is required for proteostasis, we first introduced the Q35::YFP construct and examined aggregation as these animals aged. Strikingly, unlike *sel‐12(ar131)*, *sel‐12(ok2078),* or *sel‐12(ty11)* mutants, we do not observe premature aggregation in the *sel‐12(D‐>A)* mutants (Figure 1b,c), suggesting the premature Q35::YFP aggregation observed in *sel‐12(ar131)*, *sel‐12(ok2078),* and *sel‐12(ty11)* mutants occurs independent of gamma‐secretase activity. Next, we examined the expression of human Abeta1‐42 in *sel‐12(D‐>A)* mutants. Again, in contrast to the *sel‐12(ar131), sel‐12(ok2078),* or *sel‐12(ty11)* mutants, we did not observe exacerbated swimming defects in the *sel‐12(D‐>A)* mutants expressing Abeta1‐42 (Figure 2a; Figure S1a). Moreover, analysis of *sel‐12(D‐>A)* mutants pan‐neuronally expressing human V337M mutant tau did not show an enhanced motility defect, unlike *sel‐12(ar131)*, *sel‐12(ok2078),* or *sel‐12(ty11)* mutants (Figure 2b; Figure S1b). Lastly, to investigate endogenous proteostasis after heat stress, we exposed *sel‐12(D‐>A)* mutants to 37°C for 1–4 hr and measured survival. Markedly, in contrast to *sel‐12(ar131), sel‐12(ok2078),* and *sel‐12(ty11)* mutants, the *sel‐12(D‐>A)* mutants displayed similar survival as wild‐type animals (Figure 1e). Since *C. elegans* expresses an additional presenilin homolog, HOP‐1, that can act redundantly with SEL‐12 to mediate Notch signaling (Li & Greenwald, 1997), we tested whether HOP‐1 has a role in proteostasis. To accomplish this, we utilized a gamma‐secretase inhibitor, compound E, that specifically inhibits SEL‐12 mediated gamma‐secretase activity (Francis et al., 2002;Sarasija & Norman, 2015) to treat *hop‐1* null mutants and examined the accumulation of Q35 aggregates in these animals. While these animals showed a clear defect in Notch signaling indicating disruption of gamma‐secretase activity, unlike *sel‐12(ar131)*, *sel‐12(ok2078),* or *sel‐12(ty11)* mutants, we did not observe premature aggregation of Q35 day 3 adult (Figure S3). Together, these data indicate that loss of gamma‐secretase activity is not triggering the collapse of proteostasis in *sel‐12* mutants.

### 2.3 Reduction in ER to mitochondrial calcium signaling prevents the collapse of proteostasis in *sel‐12* mutants

**Figure 3:**
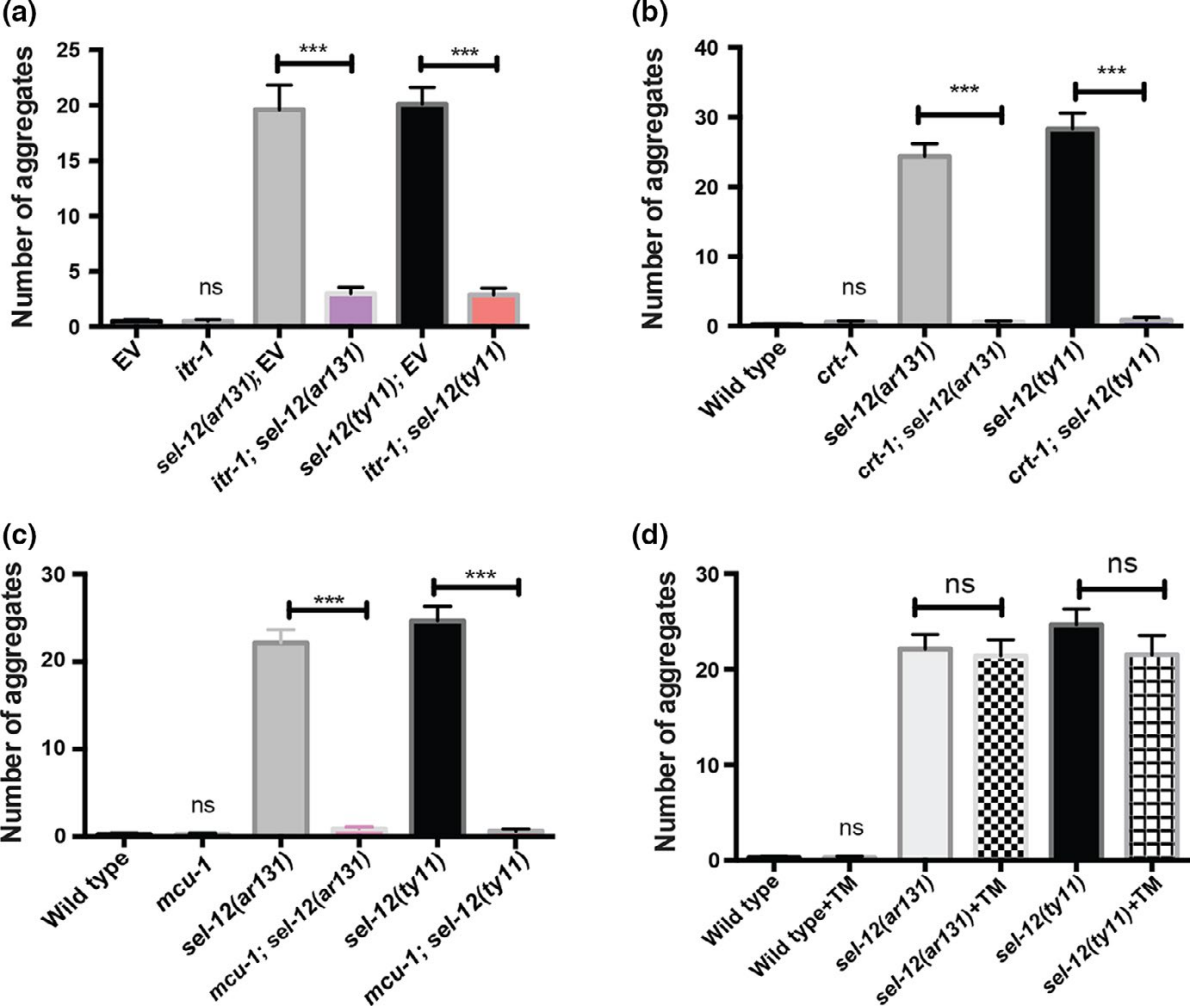
Reduction in ER‐mitochondrial Ca^2+^ signaling in *sel‐12* mutants rescues premature polyQ aggregation. (a) Quantification of Q35::YFP aggregation in day 3 adult wild‐type, *sel‐12(ar131),* and *sel‐12(ty11)* animals grown on RNAi plates seeded with either empty vector (EV) or *itr‐1(RNAi)* (*N* = 30 animals per group). (b) Quantification of Q35::YFP aggregation in wild‐type, *sel‐12(ar131), sel‐12(ty11)*, *crt‐1(jh101); sel‐12(ar131)* and *crt‐1(jh101); sel‐12(ty11)* mutant animals at day 3 adulthood (*N* = 25 animals per group). (c) Quantification of Q35::YFP aggregation in wild‐type, *sel‐12(ar131), sel‐12(ty11)*, *mcu‐1(tm6026); sel‐12(ar131)* and *mcu‐1(tm6026); sel‐12(ty11)* at day 3 adulthood (*N* = 25 animals per group). (d) Quantification of Q35::YFP aggregation in wild‐type, *sel‐12(ar131)* and *sel‐12(ty11)* animals grown in the absence or presence of 10 μg/ml tunicamycin at day 3 adulthood (*N* = 20 animals per group). Data are displayed as mean ± *SEM*, and all comparisons have been made to wild‐type animals or indicated strains. ns *p* > .05, **p < .01, and ***p < .001 were determined using one‐way ANOVA

Since the protease activity of SEL‐12 is not contributing to proteostasis, we next sought to identify the possible mechanism leading to the collapse of proteostasis in *sel‐12* mutants. Previously, we found that *sel‐12* mutants have elevated ER to mitochondrial calcium transfer, which leads to mitochondrial dysfunction and neurodegeneration (Figure S2) (Sarasija et al., 2018;Sarasija & Norman, 2015). Importantly, we found that reducing ER calcium release or mitochondrial calcium uptake could restore mitochondrial activity and rescue neurodegeneration in *sel‐12* mutants. Therefore, we investigated whether reducing ER to mitochondrial calcium signaling could protect against the proteostatic collapse observed in *sel‐12* mutants. First, we used RNA interference to knockdown inositol 1,4,5 trisphosphate receptor function [*itr‐1(RNAi)*] and investigated the premature aggregation of Q35::YFP in *sel‐12* mutants. Strikingly, we found that reducing ER calcium release by *itr‐1(RNAi)* prevented the premature aggregation of Q35::YFP that occurs in day 3 adult *sel‐12* mutants (Figure 3a). To further implicate the role of ER calcium release in promoting premature aggregation of Q35::YFP in *sel‐12* mutants, we introduced a calreticulin (*crt‐1*) null mutation into the *sel‐12* mutant background and analyzed Q35::YFP distribution. Calreticulin is an ER calcium‐binding protein and when mutated has been shown to reduce ER calcium release (Michalak, Corbett, Mesaeli, Nakamura, & Opas, 1999;Sarasija et al., 2018;Xu, Tavernarakis, & Driscoll, 2001). Similar to *itr‐1(RNAi)* treatment, the *crt‐1* null mutation suppresses the premature Q35::YFP aggregation in *sel‐12* mutants (Figure 3b). Since loss of *crt‐1* function is known to induce an ER unfolded protein stress response (Sakaki et al., 2012), we investigated whether activation of ER unfolded protein stress response by treatment with tunicamycin can prevent the premature aggregation of Q35::YFP in *sel‐12* mutants (Figure S4). Thus, we treated *sel‐12* mutants with tunicamycin and examined Q35::YFP aggregation. Unlike *itr‐1(RNAi)* treatment or loss of *crt‐1*, tunicamycin‐treated *sel‐12* mutants showed premature aggregation of Q35::YFP similar to vehicle‐treated *sel‐12* mutants (Figure 3d).

To further investigate whether ER to mitochondrial calcium signaling is pivotal in causing the collapse of proteostasis in *sel‐12* mutants, we next tested whether reducing mitochondrial calcium uptake could suppress premature Q35::YFP aggregation in *sel‐12* mutants. To accomplish this, we utilized a mitochondrial calcium uniporter (*mcu‐1*) null mutation, which we and others have shown results in reduced mitochondrial calcium levels (Sarasija et al., 2018;Xu & Chisholm, 2014). Thus, we introduced the *mcu‐1* null mutation into *sel‐12* mutants and examined Q35::YFP distribution. Consistent with the notion that ER to mitochondrial calcium signaling is leading to proteostatic collapse in *sel‐12* mutants, we observed a suppression of premature Q35::YFP aggregation in *mcu‐1, sel‐12* double mutants (Figure 3c). Moreover, we found that loss of *mcu‐1* does not lead to an ER stress response (Figure S4a,b). Interestingly, while reducing ER to mitochondrial calcium signaling suppresses the premature Q35::YFP aggregation in day 3 adult *sel‐12* mutants, it is not able to prevent aggregation in older animals (Figure S5), suggesting disruption of ER‐mitochondrial homeostasis is a critical flaw in *sel‐12* mutants that leads to premature proteostatic collapse.

**Figure 4:**
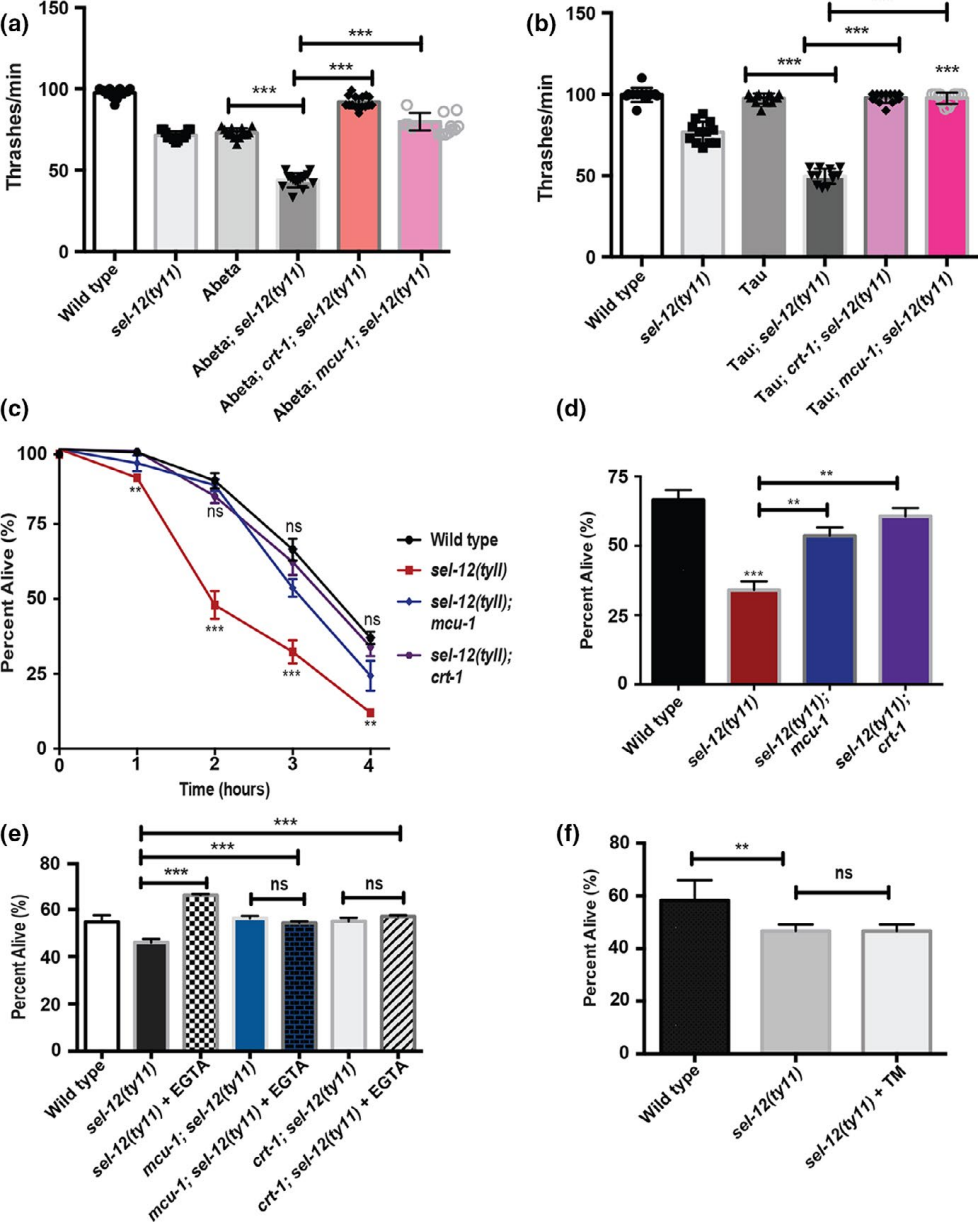
Reduction in ER‐mitochondrial calcium signaling suppresses proteostatic collapse in *sel‐12* mutants. (a) Quantification of swimming behavior of wild‐type, *sel‐12(ty11), crt‐1(jh101); sel‐12(ty11)* and *mcu‐1(tm6026); sel‐12(ty11)* mutant animals expressing human Abeta1‐42 (*dvIs100*) was conducted by measuring the number of body bends in liquid (20 animals per group). (b) Quantification of swimming behavior of wild‐type, *sel‐12(ty11)*, *crt‐1(jh101); sel‐12(ty11)* and *mcu‐1(tm6026); sel‐12(ty11)* mutant animals expressing human V337M mutant tau (*bkIs10*) was conducted by measuring the number of body bends in liquid (*N* = 20 animals per group). (c) Survival of day 1 adult animals after exposure to heat stress at 37°C for 1–4 hr (50 animals per group, done in triplicate). (d) Survival of day 1 adult animals after exposure to heat stress at 37°C for 3 hr (50 animals per group, done in triplicate). (e) Survival of day 1 adult animals treated with EGTA after exposure to heat stress at 37°C for 3 hr (30 animals per group, done in triplicate). (f) Survival of day 1 adult animals treated with 10 μg/ml tunicamycin after exposure to heat stress at 37°C for 3 hr (30 animals per group, done in triplicate). Data are displayed as mean ± *SEM*, and all comparisons have been made to wild‐type animals or indicated strains. ns *p* > .05, **p < .01, and ***p < .001 were determined using one‐way ANOVA (a, b, d, e, and f) and two‐way ANOVA (c)

To further examine the role of ER‐mitochondrial calcium signaling in proteostasis, we next investigated whether the same genetic manipulations could also suppress the enhanced locomotion defects observed in *sel‐12* animals expressing human Abeta1‐42 or human V337M mutant tau. Similarly, we found that reducing ER calcium release using *crt‐1* mutants or mitochondrial calcium uptake using *mcu‐1* mutants rescued the exacerbated swimming defects observed in *sel‐12* mutants expressing Abeta1‐42 and V337M mutant tau (Figure 4a,b). Lastly, we investigated whether altered ER to mitochondrial calcium signaling leads to proteostatic collapse in *sel‐12* mutants after extreme heat stress. We exposed *crt‐1; sel‐12* and *mcu‐1;* and *sel‐12* double mutants to 37°C, and determined their survival. Unlike *sel‐12* mutants alone, *crt‐1; sel‐12* and *mcu‐1;* *sel‐12* double mutants were significantly better at surviving extreme heat stress (Figure 4c,d). Moreover, unlike tunicamycin, we found that supplementing *sel‐12* mutants with the calcium chelator, EGTA, could also improve the survival of *sel‐12* mutants to extreme heat stress (Figure 4e,f). Collectively, these data demonstrate that defective ER‐mitochondrial calcium homeostasis is promoting the collapse of proteostasis in *sel‐12* mutants.

### 2.4 Antioxidant supplementation suppresses proteostatic collapse in *sel‐12* mutants

**Figure 5:**
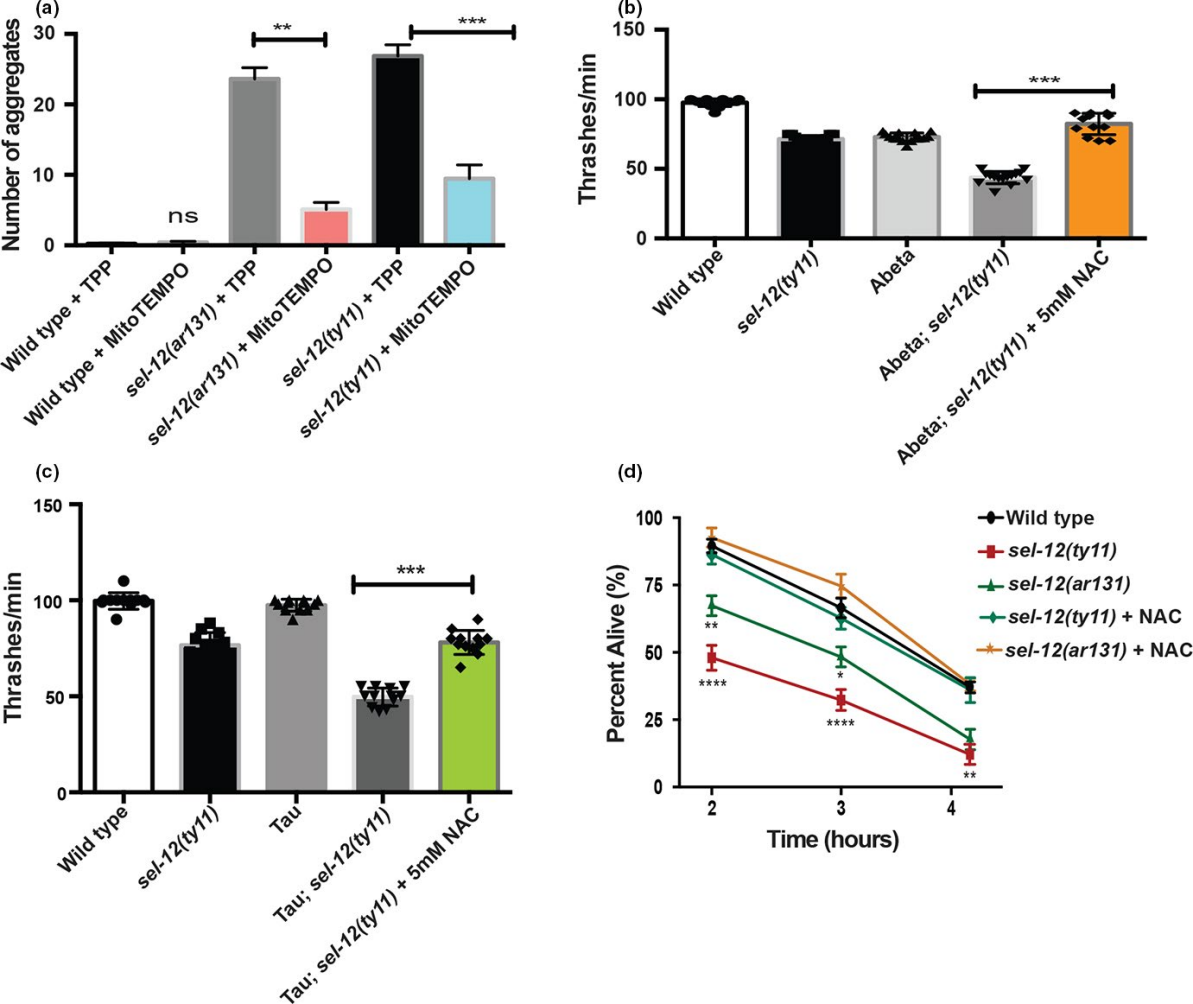
Antioxidant supplementation protects against proteostatic collapse in *sel‐12* mutants. (a) Quantification of Q35::YFP aggregation of wild‐type, *sel‐12(ar131)*, *sel‐12(ty11*) day 3 adult animals after exposure to TPP and MitoTEMPO (*N* = 20 animals per group). (b) Swimming assay was scored in wild‐type and *sel‐12(ty11)* day 1 adult animals expressing human Abeta1‐42 treated with 5 mM NAC by measuring the number of body bends in liquid (*N* = 20 animals in each group). (c) Swimming assay were scored in wild‐type and *sel‐12(ty11)* day 1 adult animals expressing human V337M mutant tau treated with 5 mM NAC by measuring the number of body bends in liquid (*N* = 20 animals in each group). (d) Survival of day 1 adult wild‐type, *sel‐12(ar131),* and *sel‐12(ty11)* mutant animals treated with 5 mM NAC after exposure to heat stress at 37°C for 2–4 hr (50 animals per group, done in triplicate). Data are displayed as mean ± *SEM*, and all comparisons have been made to wild‐type animals or indicated strains. ns *p* > .05, **p < .01, and ***p < .001 were determined using one‐way ANOVA (a, b, and c) and two‐way ANOVA (d)

Since calcium is known to stimulate mitochondrial respiration (Llorente‐Folch et al., 2015;Tarasov, Griffiths, & Rutter, 2012) and we previously demonstrated that elevated ER to mitochondria calcium signaling in *sel‐12* mutants leads to increased mitochondrial reactive oxygen species (ROS) production via electron leak caused by increased mitochondrial respiration (Sarasija et al., 2018), we sought to determine whether the proteostasis defects observed in *sel‐12* mutants is caused by oxidative stress, a known inducer of proteostasis dysfunction (Korovila et al., 2017). Consistent with this notion, we previously found that antioxidant supplementation prevented *sel‐12* neurodegenerative phenotypes (Sarasija et al., 2018). Thus, these data implicate ROS as causing neurodegeneration in *sel‐12* mutants. To investigate the role ROS has in the collapse of proteostasis in *sel‐12* mutants, we treated *sel‐12* mutants with antioxidants and examined proteostasis defects. First, we tested whether the mitochondrial‐targeted superoxide scavenger MitoTEMPO or TPP (triphenylphosphonium), which lacks the superoxide scavenger moiety, could prevent the premature Q35::YFP aggregation. Consistent with our previous analyses of neurodegeneration in *sel‐12* mutants, we found that MitoTEMPO but not TPP could prevent the premature Q35::YFP aggregation in *sel‐12* mutants (Figure 5a). Interestingly, MitoTEMPO did not prevent aggregation in older *sel‐12* mutant or wild‐type animals (Figure S2), suggesting oxidative stress is a crucial weakness in *sel‐12* mutants that leads to premature proteostatic collapse. To further investigate the role of ROS in proteostasis in *sel‐12* mutants, we examined if antioxidant supplementation could also suppress the exacerbated locomotion defects observed in *sel‐12* animals expressing human Abeta1‐42 or human V337M mutant tau. To do this, we treated *sel‐12* transgenic animals with a widely used antioxidant *N*‐acetylcysteine (NAC) and measured swimming activity. Similar to MitoTEMPO treatment, we found that NAC could rescue the aggravated swimming defects observed in *sel‐12* animals expressing human Abeta1‐42 or mutant tau (Figure 5b,c). Lastly, we tested if NAC treatment could rescue the proteostatic collapse that occurs in *sel‐12* mutants after extreme heat stress. We found that *sel‐12* mutants supplemented with 5 mM NAC could survive significantly better than vehicle‐treated animals (Figure 5d). Taken together, these data indicate that elevated ROS in the *sel‐12* mutants is promoting proteostatic collapse.

**Figure 6:**
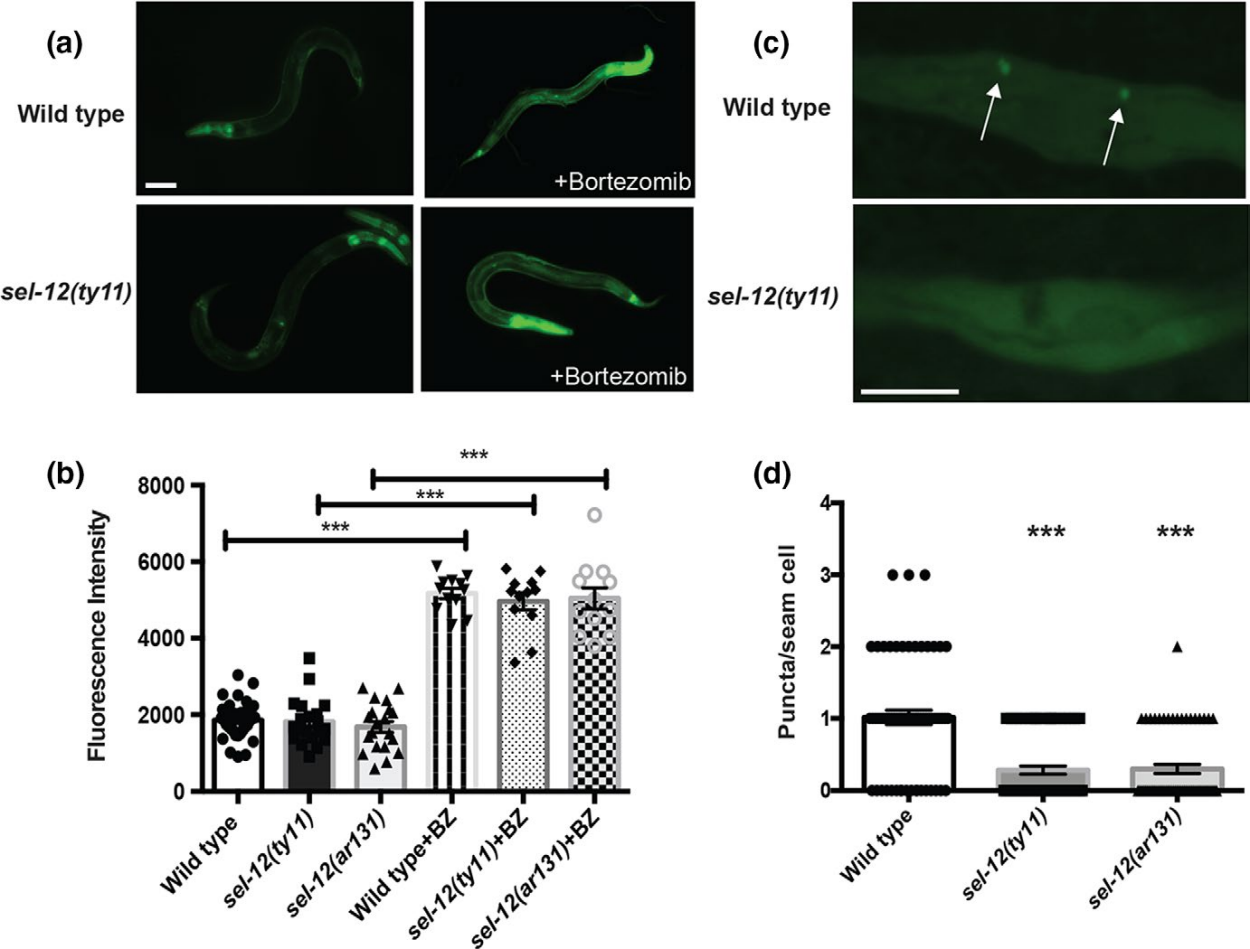
*sel‐12* mutants show reduced autophagosome formation. (a) Representative images of wild‐type and *sel‐12(ty11)* animals expressing *rpt‐3p*::GFP (*mgIs72*) treated with proteasome inhibitor (bortezomib) for 24 hr at day 1 adulthood. Scale bar equals 100 μm. (b) Quantification of *rpt‐3p*::GFP intensity in wild‐type, *sel‐12(ty11)* and *sel‐12(ar131*) animals. (c) Representative images of wild‐type and *sel‐12(ty11)* animals expressing the autophagosome marker *lgg‐1p::lgg‐1::*GFP (*adIs2122*). The arrows indicate autophagosome formation (LGG‐1::GFP puncta). Scale bar equals 10 μm. (d) Quantification of LGG‐1::GFP puncta in wild‐type, *sel‐12(ty11),* and *sel‐12(ar131)* animals expressing *lgg‐1p::lgg‐1::*GFP (*adIs2122*) at day 1 of adulthood. Data are displayed as mean ± *SEM*, and all comparisons have been made to wild‐type animals or indicated strains. ns *p* > .05 and ***p < .001 were determined using one‐way ANOVA

While oxidative stress is playing a decisive role in the collapse of proteostasis in *sel‐12* mutants, we investigated two vital proteostasis pathways involved in protein degradation, the ubiquitin‐proteasome system, and autophagy‐lysosome system, in *sel‐12* mutants to determine whether either pathway are encumbered in *sel‐12* mutants. First to investigate proteasome activity, we utilized a GFP reporter, *rpt‐3p*::GFP, that is activated upon proteasome dysfunction. Using this reporter, it has been shown that knockdown of proteasomal components or using proteasome inhibitors stimulates GFP expression and has been used to gauge proteasome function (Lehrbach & Ruvkun, 2016;Li et al., 2011). Analysis of this reporter in *sel‐12* mutants shows similar expression to wild‐type animals suggesting normal basal activity of the proteasome in *sel‐12* mutants (Figure 6a,b). To test whether *sel‐12* mutants can respond to proteasome disruption, we treated animals with the proteasome inhibitor bortezomib. Treatment with bortezomib resulted in similar activation of *rpt‐3p*::GFP in *sel‐12* mutants as wild‐type animals (Figure 6a,b). These data suggest that *sel‐12* mutants have normal proteasome function. Next, we analyzed the formation of autophagosomes in *sel‐12* mutants and wild‐type animals. To accomplish this, we employed an autophagosome marker encoded by the *lgg‐1* gene. *lgg‐1* encodes an Atg8/LC3 homolog that decorates the autophagosome and is utilized as marker of autophagosome formation (Kang, You, & Avery, 2007;Meléndez et al., 2003). Analysis of LGG‐1::GFP in day 1 adults revealed a slight but significant decrease in autophagosome number in *sel‐12* mutants compared with similarly aged wild‐type animals (Figure 6c,d). These data suggest that *sel‐12* mutants have reduced autophagy and this along with the mitochondrial generated oxidative stress likely exacerbates the collapse of proteostasis observed in *sel‐12* mutants.

## 3 DISCUSSION

Our finding that mutations in *sel‐12* result in proteostatic collapse due to elevated ER‐mitochondrial calcium signaling and ROS production implicates a critical function of presenilin in mediating ER‐mitochondrial calcium homeostasis and when presenilin function is disrupted this leads to mitochondrial dysregulation, which promotes a proteotoxic environment leading to the collapse of proteostasis. While altered presenilin function has been known to have a role in AD for over 20 years, the functional consequences of presenilin mutations are not understood (Selkoe & Hardy, 2016). Presenilins are an ancient conserved protein family that are found on endomembranes in plants, amoeba, invertebrates, and mammals, including humans (Levitan & Greenwald, 1998;Smolarkiewicz et al., 2013). However, their function on endomembranes has not been explored in great detail. In animals, presenilins have been localized to the ER and are concentrated at ER‐mitochondrial contacts (Area‐Gomez et al., 2009;De Strooper et al., 2012;Levitan & Greenwald, 1998). Our data using *C. elegans* indicate a critical role of SEL‐12/presenilin in regulating ER‐mitochondrial calcium homeostasis and loss of presenilin results in an increase in mitochondrial calcium loading, a concomitant intensification in oxidative phosphorylation and electron leak leading to an elevation in ROS (Sarasija et al., 2018). Similar to what we have observed in *C. elegans*, it has been shown previously in human cells isolated from patients with AD that mutations in presenilin result in increased oxidative phosphorylation and an elevation in ROS (Oksanen et al., 2017;Sarasija et al., 2018). Together these studies and our present study indicate a critical role of presenilin in ER‐mitochondrial homeostasis.

Since presenilin is the catalytic subunit of the gamma secretase, which cleaves the amyloid precursor protein to produce Abeta peptides, the central hypothesis regarding presenilin function in causing AD has focused on its role as an integral membrane protease. Using *C. elegans* as a model to study presenilin function enables us to explore the role of presenilin in the absence of Abeta peptide production, since the *C. elegans* genome does not encode an Abeta like peptide or encode a beta‐secretase required for Abeta production (Daigle & Li, 1993;McColl et al., 2012). Thus, the proteostasis defects and the neurodegeneration previously observed in *sel‐12* mutants (Sarasija et al., 2018) are due to defects associated with the loss of presenilin function and not due to Abeta production. Moreover, we have found that a protease dead version of presenilin does not show defects in proteostasis unlike other *sel‐12* mutations. Further blocking presenilin protease activity either genetically or pharmacologically in *C. elegans* does not cause neurodegenerative phenotypes as is observed in *sel‐12* mutants (Sarasija et al., 2018). These data suggest that presenilin dysfunction can lead to proteostasis defects and neurodegeneration in the absence of Abeta peptides and gamma‐secretase activity.

The two major pathways involved in protein degradation that are critical for proteostasis include the ubiquitin‐proteasome system and the autophagy‐lysosome system (Klaips, Jayaraj, & Hartl, 2018;Labbadia & Morimoto, 2015). Although, using the assay described here, the proteasome in *sel‐12* mutants is functioning normally, analysis of autophagosome formation is reduced in *sel‐12* mutants compared with wild‐type animals. This suggests that autophagy is encumbered in *sel‐12* mutants. While the role autophagy has in the collapse of proteostasis in *sel‐12* mutants will require further investigation, of note, several studies have implicated a role of presenilins in autophagy, although this role has remained unclear (Fedeli, Filadi, Rossi, Mammucari, & Pizzo, 2019;Lee et al., 2010;Martín‐Maestro et al., 2017;Raemaekers, Esselens, & Annaert, 2005;Reddy et al., 2016;Zhang et al., 2012).

Here, despite the defect in autophagy, we have found that reducing ER to mitochondrial calcium signaling or treatment with antioxidants can suppress the *sel‐12* mutants proteostasis defect, indicating the central role calcium homeostasis and oxidative stress have in proteostatic collapse in *sel‐12* mutants. Interestingly, we find that these treatments do not prevent proteostasis decline as wild‐type or *sel‐12* mutant animals further age. Together, these data indicate that in young adult animals loss of ER‐mitochondrial calcium homeostasis due to defective presenilin function leads to a rise in oxidative stress promoting premature proteostatic collapse, thus highlighting a critical role of presenilin in proteostasis, which may underlie the cause of neurodegeneration in FAD patients with alterations in the presenilin genes.

## 4 MATERIAL AND METHODS

### 4.1 *Caenorhabditis elegans* strains and genetics

*Caenorhabditis* *elegans* were maintained a previously described (Brenner, 1974). In brief, *C. elegans* were grown on OP50‐seeded NGM plates at 20°. Animals were synchronized for all experiments by bleaching plates containing gravid worms, and the progeny were rocked in M9 buffer for <48 hr. The synchronized L1s were then allowed to grow on NGM plates seeded with OP50 until the stage required for the experiments was reached. The following strains were used in this study: N2 is the wild‐type strain*, crt‐1(jh101) V, hop‐1(ar179) I, mcu‐1(tm6026) IV*, *sel‐12(ar131,ok2078, ty11) X, adIs2122* [*lgg‐1p::lgg‐1::GFP, dvIs100* [*unc‐54p*::Abeta‐1‐42::*unc‐54* 3′UTR], *bkIs10* [*aex‐3p*::tau–V337M, *myo‐2p*::GFP], *mgIs72* [*rpt‐3p*::GFP], *rmIs132[unc‐54p::*polyQ35::YFP*], oxIs12* [*unc‐47p::GFP*], *and takEx415* [*mec‐7p*::mito‐GCaMP6f::SL2::mCherry].

### 4.2 CRISPR/Cas9 genome engineering

CRISPR/Cas9‐induced knock‐in of *sel‐12* was carried out as previously described (Prior, Jawad, MacConnochie, & Beg, 2017). In brief, sgRNA targeting the D226 codon (synthesized by Synthego, Seq: uguuaucucgguuugggauc) was injected with purified Cas9 protein (Synthego), single‐stranded DNA repair template (synthesized by Invitrogen Seq: 5′AATGGACTGTGTGGTTTGTGCTGTTTGTTATCTCGGTTTGGGcTCTaGTTGCCGTGCTCACACCAAAAGGACCATTGAGATATTTGG 3′) and a transgenic injection marker *ttx‐3p*::GFP (kind gift from Oliver Hobert, Columbia University). From the progeny of injected animals, *ttx‐3p*::GFP‐positive animals were selected and their progeny were scored for egg‐laying defective phenotype, which indicates loss of *sel‐12*/gamma‐secretase protease activity. These animals were subjected to DNA sequencing to confirm D226A knock‐in was obtained.

### 4.3 Drug treatments

10 μg/ml of tunicamycin (Sigma) and 10 μM bortezomib (LC Laboratories) were prepared in DMSO and added to NGM media. 20 mM EGTA (RPI) was prepared in dH_2_O and added to NGM media. Synchronized animals were grown on these plates for further analysis. Tunicamycin‐treated animals were grown on tunicamycin plates for 24 hr for heat stress survival assays and 48 hr for Q35::YFP aggregation assays. Bortezomib‐treated animals were grown on bortezomib plates for 24 hr. EGTA‐treated animals were grown on EGTA plates for 24 hr.

### 4.4 Inhibition of gamma‐secretase activity

The surface of unseeded NGM plates is coated with 100 μl of 200 mM Compound E (Tocris bioscience), a gamma‐secretase inhibitor, and seeded with OP50 the next day as previously described (Sarasija & Norman, 2015). Synchronized wild‐type and *hop‐1* mutant L4‐staged animals are grown on these plates until day three adults, which are then used for analysis. Gamma‐secretase inhibition was confirmed by sterility of the *hop‐1* mutants that is induced by the loss of Notch signaling.

### 4.5 Antioxidant supplementation

Animals are moved to NGM plates containing 500 μM (2‐(2,2,6,6‐tetramethylpiperidin‐1‐oxyl‐4‐ylamino) −2‐oxoethyl) triphenylphosphonium chloride (mitoTEMPO) (Sigma‐Aldrich), 500 μM triphenylphosphonium chloride (TPP) (Sigma‐Aldrich) and 5 mM N‐acetylcysteine (NAC) (Sigma‐Aldrich) as L1 larvae. These animals are used for analyses as day 1 adults for the heat stress, Abeta1‐42, and mutant tau proteostasis analyses and day 1, 3, and 5 adults for the Q35::YFP aggregation studies.

### 4.6 RNAi interference

Age‐synchronized L1 animals were allowed to grow to adults on RNA interference (RNAi) plates seeded with empty vector, *itr‐1* RNAi‐containing bacteria. RNAi clone for *itr‐1* was obtained from Ahringer RNAi library (Kamath et al., 2003) and was verified by DNA sequencing.

### 4.7 Quantitation of polyQ aggregation

Animals were viewed using 10× objectives on a Zeiss AxioObserver microscope equipped with an Andor Clara CCD camera. Images were compiled using Metamorph software, and the number of polyQ aggregates was counted as day 1, 3, and 5 age‐synchronized adults. Aggregates were defined as discrete structures with boundaries distinguishable from surrounding fluorescence on all sides.

### 4.8 Locomotion assay

Swimming behavior was characterized as previously described (Nawa & Matsuoka, 2012). In brief, age‐synchronized day 1 adult worms were transferred to fresh unseeded plates and allowed to crawl freely to remove bacteria from worm. After which they were picked singly into wells of a plate containing 2% solidified agarose at the bottom of each well with 100 μl M9 buffer. The numbers of body bends were counted for 1 min.

### 4.9 Heat stress survival assay

Age‐synchronized worms at day 1 young adult stage were transferred to fresh agar plates with OP50 *Escherichia coli*, then exposed to heat shock by placing the plates in a 37°C water bath for 1, 2, 3, and 4 hr and then removed and allowed to recover at 20°C. Worms were considered dead if they did not display any movement in response to repeated prodding with a thin wire.

### 4.10 Proteosome and autophagosome formation assays

To investigate the state of the ubiquitin‐proteasome, we utilized the *rpt‐3p*::GFP (*mgIs72*) strain, which activates GFP express upon proteasome dysfunction (Lehrbach & Ruvkun, 2016). Synchronized day 1 adult *mgIs72* animals were imaged using a 10× objective lens on a Olympus BX61 microscope equipped with a PCO Panda sCMOS camera. Images were compiled using Metamorph software, and fluorescence intensity was quantified using Image J. To investigate the formation of autophagosomes, we utilized the *lgg‐1p::lgg‐1*::GFP (*adIs2122*) strain, which decorates autophagosomes with GFP puncta (Kang et al., 2007;Meléndez et al., 2003). Synchronized day 1 adult *mgIs72* animals were imaged using a 10× objective lens on a Zeiss Axio Observer microscope equipped with an Andor Clara CCD camera. Images were compiled using Metamorph software, and fluorescence intensity was quantified using ImageJ.

### 4.11 Mechanosensation assay

Age‐synchronized day 1 adult animals were evaluated for the ability to respond to light touch using an eyebrow hair glued to the end of a Pasteur pipette tip (Sarasija et al., 2018). Animals were scored based on their responsiveness to a total of 10 touches: five to the anterior (between the head and vulva) and five touches to the posterior (between the tail and vulva). If the animal moves forward in response to a light touch in the posterior or backward after an anterior touch, respectively, then the animal receives a score of 1 for a maximum score of 10 (100% response) or a minimum score of 0 (0% response).

### 4.12 Mitochondrial calcium concentration measurement

The mitochondrial calcium concentration in the mechanosensory neurons was measured using *takEx415* animals, expressing mec*‐7p*::mito‐GCaMP6f::SL2::mCherry. The fluorescence intensity of both the GCaMP6f (genetically encoded calcium indicator) and mCherry (used here as an expression control) was measured using a 63× objective lens on a Zeiss Axio Observer microscope and Andor Clara CCD camera and Metamorph software, as previously described (Sarasija et al., 2018). The GCaMP6 fluorescence intensity in each animal was normalized to mCherry intensity using ImageJ.

### 4.13 Oxygen consumption rate measurement

Oxygen consumption rate (OCR, indicative of mitochondrial OXPHOS) in day one sterilized animals was measured using the Seahorse XFp Extracellular Flux Analyzer (Agilent Seahorse Technologies) (Sarasija & Norman, 2019). Each assay run compared the OCR between two genotypes; in three wells each (containing 20 animals) and five measurements were made for each well for basal OCR and maximal OCR upon addition of FCCP. Assays were repeated on three separate days, and three biological replicates were analyzed.

### 4.14 Statistical analysis

GraphPad Prism software is used for statistical analysis. Student's *t* test is used only for comparing two samples and one‐way ANOVA and two‐way ANOVA with Tukey's multiple comparisons test has been used when making multiple comparisons.

## CONFLICT OF INTEREST

The authors declare no competing interests.

## AUTHOR CONTRIBUTIONS

Z.A., S.S., K.R., and K.R.N contributed to conceptualization; Z.A., S.S, K.R., and J.T.L contributed to investigation; Z.A. and K.R.N contributed to writing; K.R.N. contributed to supervision; K.R.N. contributed to funding acquisition.

## Data Availability

Strains and other reagents are available upon request.
