# Supplementary figures and images for: Corrupted ER‐mitochondrial calcium homeostasis promotes the collapse of proteostasis
Source: Aging Cell. 2019 Nov 12;19(1):e13065. doi: 10.1111/acel.13065 (PMC6974732; doi:10.1111/acel.13065)

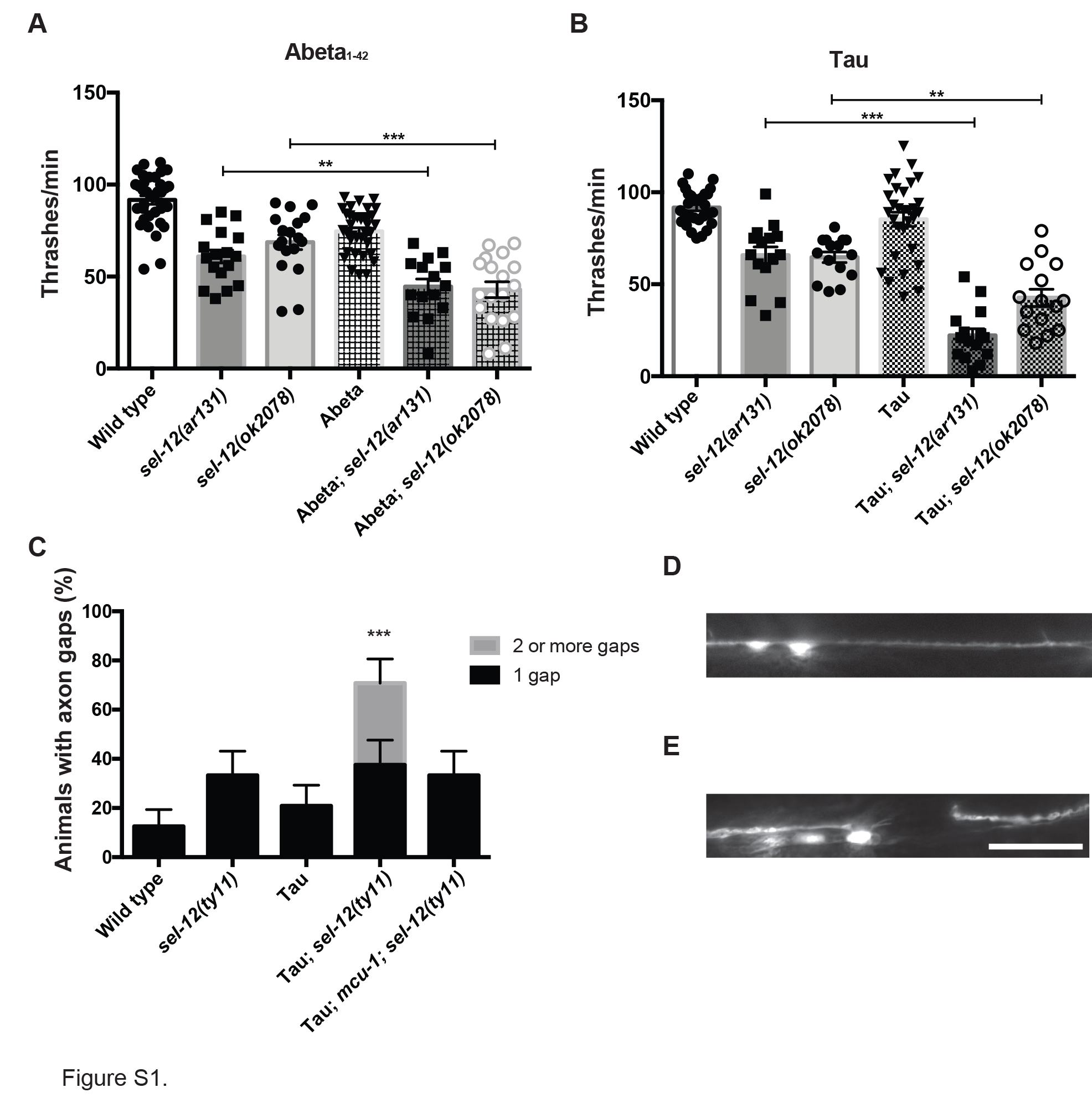

Supplement: Supplementary file 1 [file ACEL-19-e13065-s001.tif]

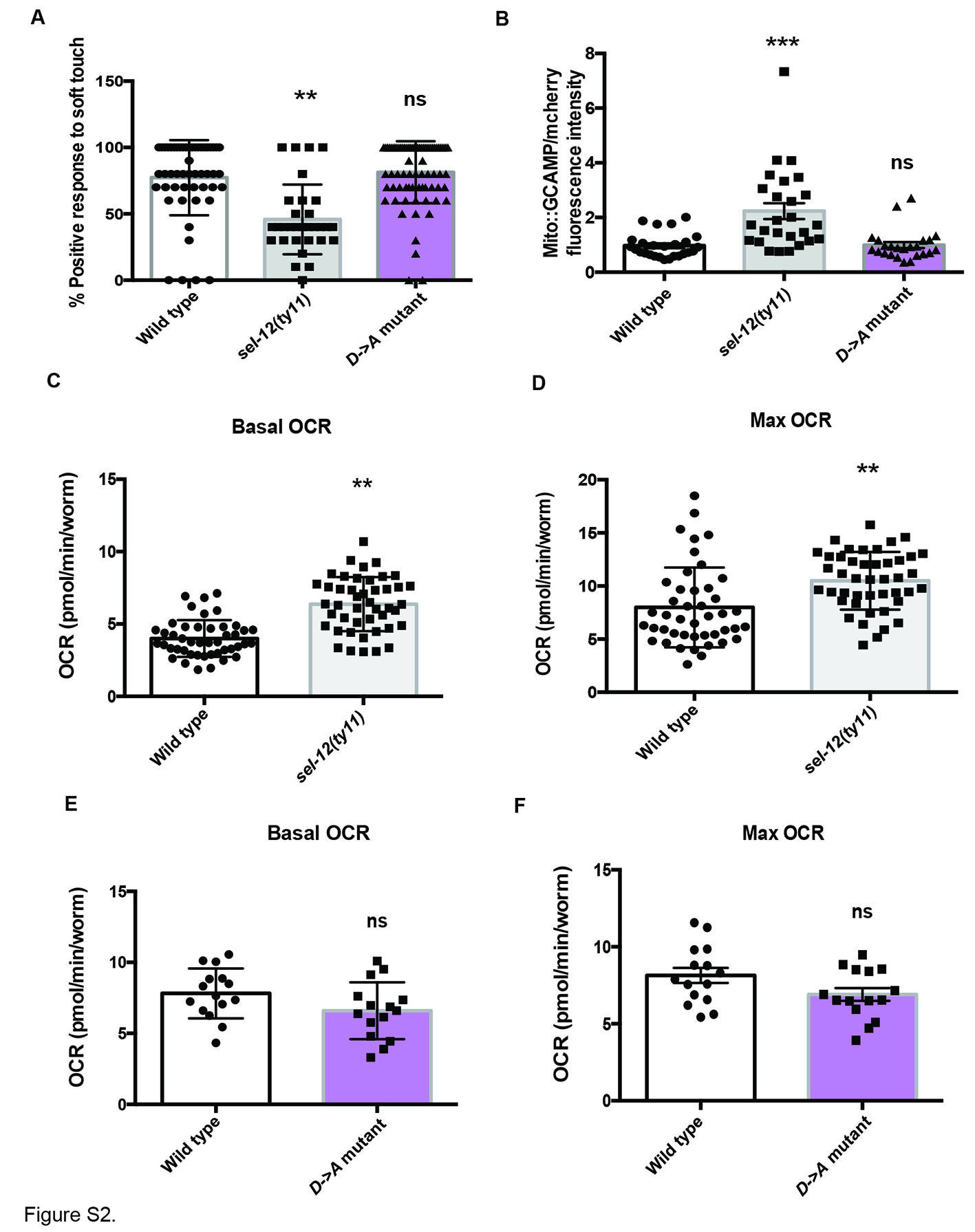

Supplement: Supplementary file 2 [file ACEL-19-e13065-s002.tif]

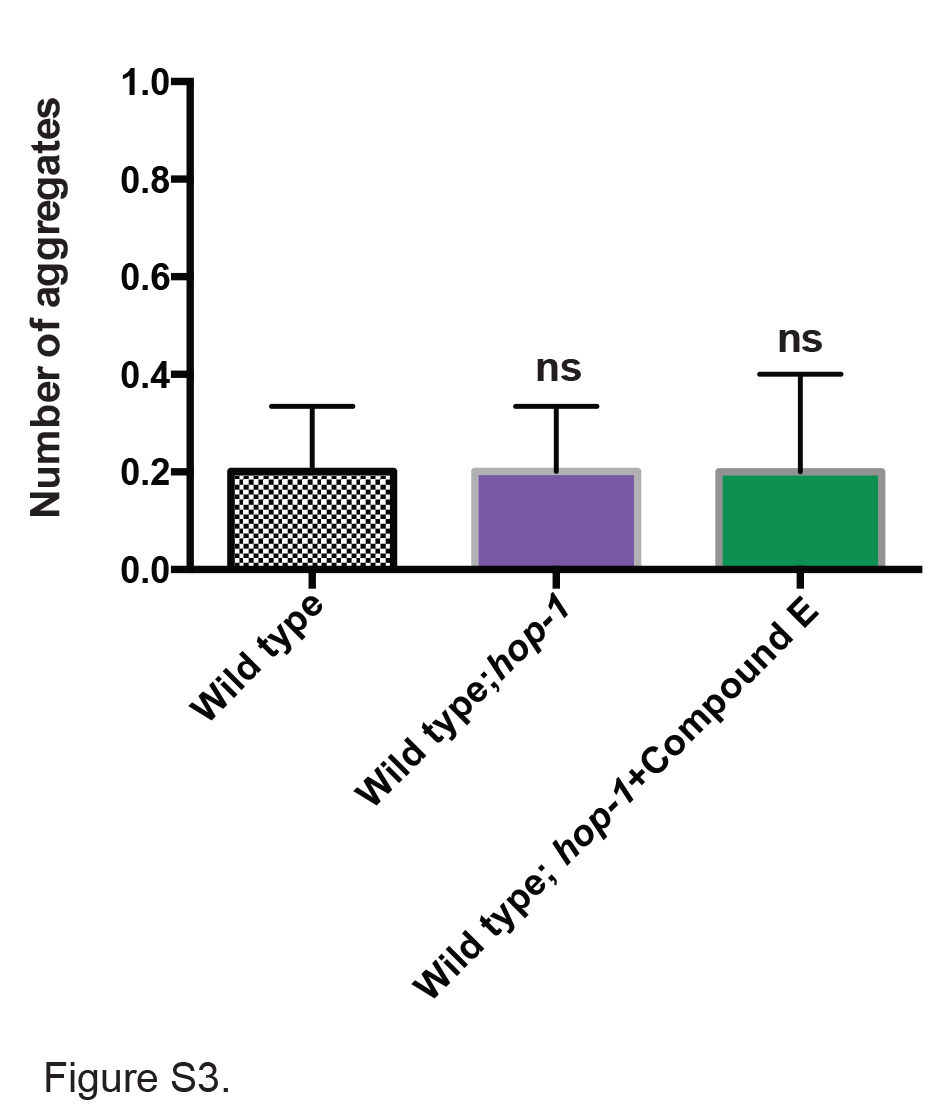

Supplement: Supplementary file 3 [file ACEL-19-e13065-s003.tif]

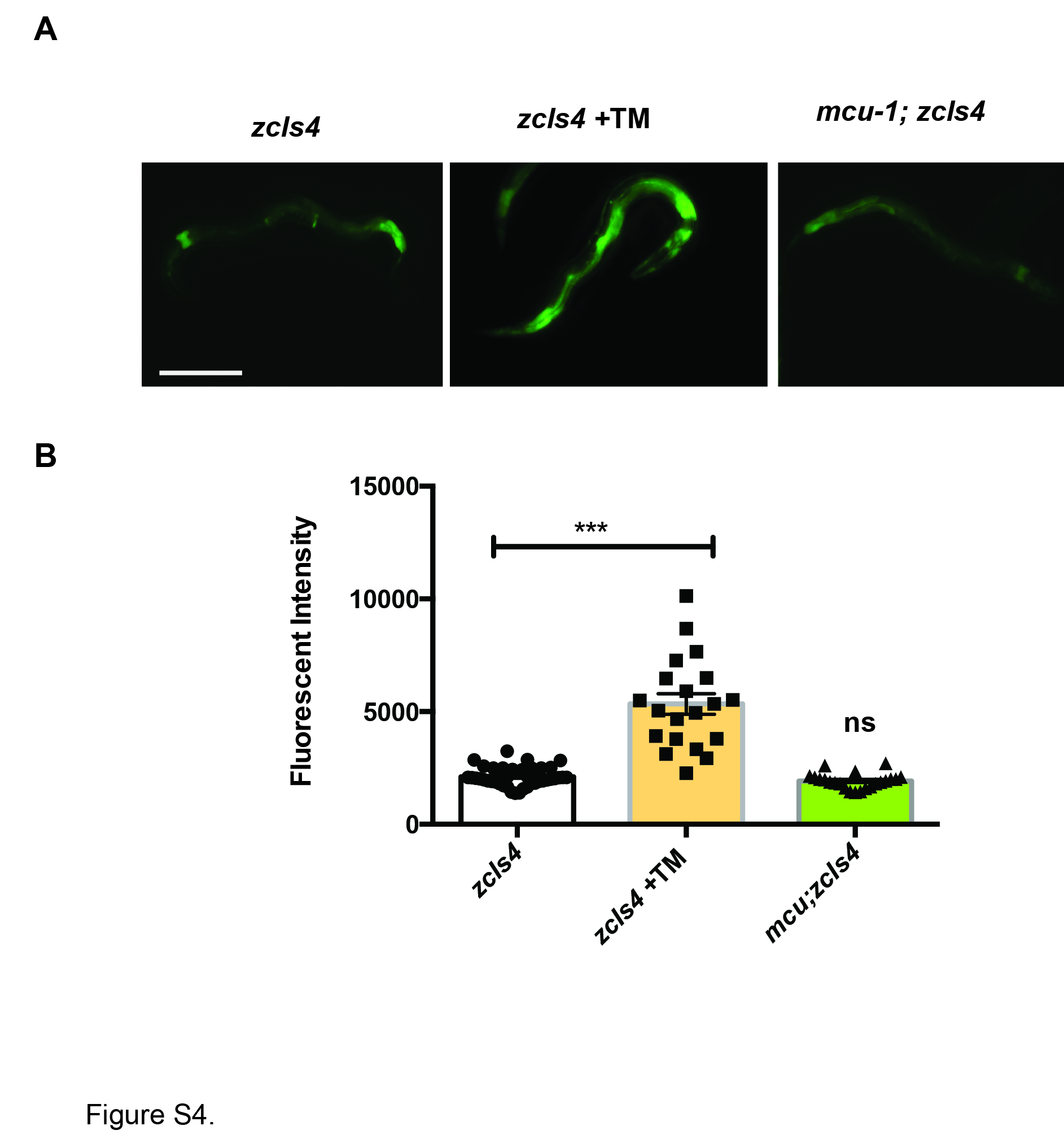

Supplement: Supplementary file 4 [file ACEL-19-e13065-s004.tif]

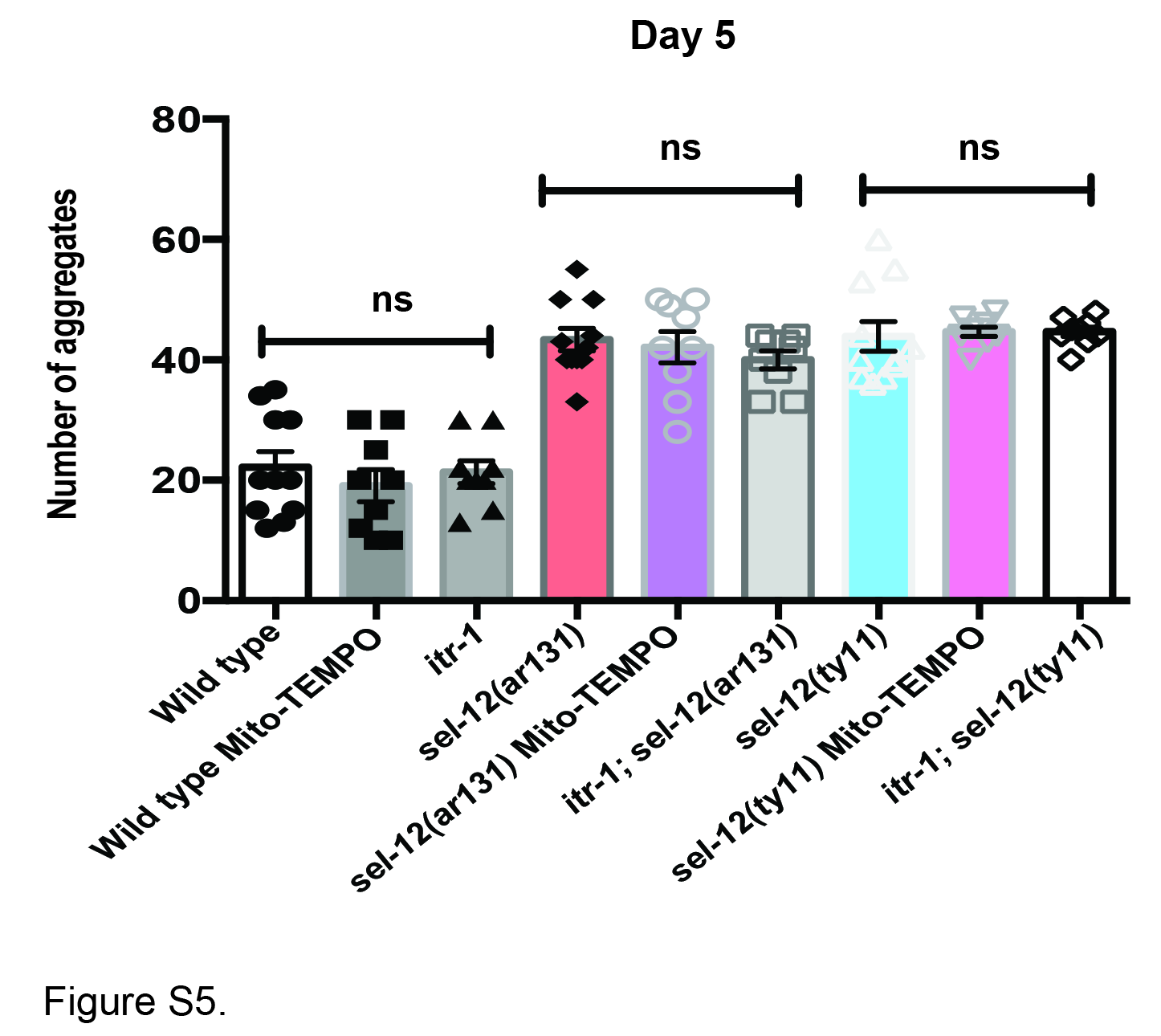

Supplement: Supplementary file 5 [file ACEL-19-e13065-s005.tif]
