# Supplementary material for: Corrupted ER‐mitochondrial calcium homeostasis promotes the collapse of proteostasis
Source: Aging Cell. 2019 Nov 12;19(1):e13065. doi: 10.1111/acel.13065 (PMC6974732; doi:10.1111/acel.13065)
Supplement: Supplementary file 6 [file ACEL-19-e13065-s006.docx]

**Supplementary Figure legends**

**Figure S1. *sel-12* mutants show exacerbated proteostasis defects due to Abeta1-42 or mutants tau expression.**

A) Swimming assay of wild-type, *sel-12(ar131)*, and *sel-12(ok2078)* animals expressing human Abeta1-42 (*dvIs100*) were conducted by measuring the number of body bends in liquid (N>15 animals per group). B) Swimming assay of wild-type, *sel-12(ar131)*, and *sel-12(ok2078)* animals expressing human mutant tau (*bkIs10*) were conducted by measuring the number of body bends in liquid (N>15 animals per group). C) Quantification of axon gaps that form in animals expressing soluble GFP in the GABAergic motor neurons (*oxIs12*). D) Representative image of the ventral nerve cord from wild-type and E) tau expressing *sel-12(ty11)* animals. Scale bar equals 5 μm. Data are displayed as mean ± SEM and all comparisons have been made to wild-type animals or indicated strains. ns p>0.05, ** p<0.01 and *** p<0.001 were determined using one-way ANOVA.

**Figure S2. Neuronal and mitochondrial dysfunction in *sel-12* mutants occurs independent of gamma-secretase protease activity.**

A). Quantification of the response of wild-type, *sel-12(ty11)* and *sel-12(D->A)* mutant animals to light anterior and posterior touch at day 1 of adulthood (N=40) B). Quantification of mitochondrial calcium in the mechanosensory neurons in wild-type, *sel-12 (ty11)* and *sel-12(D->A)* mutant animals at day 1 of adulthood (N=30 animals per group). C) Quantification of basal and E) maximal oxygen consumption rates of wild-type and *sel-12(ty11)* mutant animals at day 1 of adulthood. D) Quantification of basal and F) maximal oxygen consumption rates of wild-type and *sel-12(D->A)* mutant animals at day 1 of adulthood. Data are displayed as mean ± SEM and all comparisons have been made to wild-type animals. ns p>0.05, ** p<0.01, and *** p<0.001 were determined using one-way ANOVA (A and B) and Student’s t test (C-F).

**Figure S3. Gamma-secretase activity is not required to prevent premature Q35::YFP aggregation.**

Quantification of aggregation Q35::YFP in wild-type and hop-1 treated with gamma-secretase inhibitor (compound E) at day 3 adulthood (N=20 animals per group). Data are displayed as mean ± SEM and all comparisons have been made to wild-type animals. ns p>0.05 was determined using one-way ANOVA.

**Figure S4. *mcu-1* mutants do not induce the ER unfolded protein stress response.**

A) Representative images of *hsp-4p*::GFP (*zcIs4*) expression in wild-type (zcIs4), tunicamycin-treated (wild-type + TM) and *mcu-1* animals. Scale bars equals 200 μm. B) Quantification of *hsp-4p*::GFP intensity in wild-type, tunicamycin-treated and *mcu-1* animals. Data are displayed as mean ± SEM and all comparisons have been made to wild-type animals or indicated strains. ns p>0.05, and *** p<0.001 were determined using one-way ANOVA.

**Figure S5. Reduction in ER calcium release or antioxidant treatment does not prevent normal age-related decline in proteostasis.**

Quantification of Q35::YFP aggregation of wild-type, *sel-12(ar131)*, and *sel-12(ty11*) at day 5 adult animals after treatment of with MitoTEMPO or *itr-1*(RNAi) (N>10 animals per group). Data are displayed as mean ± SEM. ns p>0.05 for indicated strains using one-way ANOVA.
